# First report of MUTYH-associated polyposis with c.1353_1355del and c.452A>G mutations in Tolima Grande region from Colombia: a case report

**DOI:** 10.3389/fonc.2026.1873371

**Published:** 2026-08-17

**Authors:** Mabel Bohórquez-Lozano, John Suarez-Olaya, Andrea Catalina Rubio-Vargas, Fabian Castro-Valencia, Danna Diaz-Carmona, Juan Felipe Moncada-Jiménez, Jorge M. Castro, Gilbert Mateus, Angel Criollo-Rayo, Diego Buitrago, María Magdalena Echeverry de Polanco, Luis Carvajal-Carmona

**Affiliations:** 1 Grupo de Citogenética, Filogenia y Evolución de Poblaciones, Facultades de Ciencias y Ciencias de la Salud, Universidad del Tolima, Ibagué, Colombia; 2 Grupo de Biología molecular y genética, Centro de Investigación Multidisciplinario Avanzado en Salud y Nuevas Tecnologías del Eje Cafetero (CIMASTEC), Clínica Internacional de Alta Tecnología en Cáncer - CLINALTEC-Ibagué, Ibagué, Colombia; 3 Hospital Federico Lleras Acosta, Ibagué, Colombia; 4 Grupo Cirugía Gastrointestinal, Centro de Investigación Multidisciplinario Avanzado en Salud y Nuevas Tecnologías del Eje Cafetero (CIMASTEC), Clínica Internacional de Alta Tecnología en Cáncer - CLINALTEC-Ibagué, Ibagué, Colombia; 5 Genome Center, UC-Davis, Davis, CA, United States

**Keywords:** Colombian population, colorectal polyposis, germline mutations, molecular screening, *MUTYH*-associated polyposis

## Abstract

**Introduction:**

The *MUTYH* gene encodes a protein involved in DNA repair and is known for *MUTYH*-associated polyposis (MAP), a rare autosomal recessive condition that predisposes individuals to colorectal cancer (CRC), colorectal polyps and familial colorectal cancer syndrome.

**Case report:**

We describe the first Tolima Grande region from a Colombian report of individuals carrying pathogenic *MUTYH* variants c.452A>G and c.1353_1355del associated with polyposis phenotypes. Three main cases with detailed histopathological findings and family history are presented. Additionally, independent findings from Clinaltec identified three further individuals carrying c.452A>G and one heterozygous carrier of c.1353_1355del detected during predictive multigene panel testing.

**Discussion:**

These cases highlight the diagnostic and clinical challenges of distinguishing biallelic pathogenic *MUTYH* variants, which define MAP and confer high CRC risk, from monoallelic carriers, whose cancer risk is substantially lower. Misclassification may result in inappropriate surveillance strategies and missed opportunities for early detection. From a public health perspective, these findings emphasize persistent gaps in hereditary CRC prevention in underrepresented populations, including fragmented cancer registries and limited incorporation of genetic and family history data into clinical decision-making.

**Conclusion:**

This report provides evidence in Colombia of polyposis-associated pathogenic *MUTYH* variants c.452A>G and c.1353_1355del, underscoring the importance of expanding genetic evaluation for hereditary CRC in Latin American populations.

## 1 Introduction

MUTYH-associated polyposis (MAP) is an autosomal recessive familial disease associated with germline biallelic pathogenic and likely pathogenic (P/LP) variants in the *MUTYH* gene, located in the short arm of chromosome 1p34 (OMIM 604933) (1), first described in 2002 (2). *MUTYH* encodes a DNA glycosylase, involved in the base excision repair (BER) pathway, which is responsible for correcting oxidative DNA damage (3). Dysfunction of the BER pathway results in an increased rate of genetic transversions G:C > T:A in key oncogenes and tumor suppressor genes, including *APC* and *KRAS*, which has been proposed as a mutational signature of MAP. This mechanism has been used to explain how constitutional P/LP variants of *MUTYH* represent between 30% and 40% of adenomatous polyposis cases in which a constitutional variant is not detected in *APC* (4).

According to reports, the prevalence of carriers of heterozygous germline P/LP variants in *MUTYH* is 1% to 2% in populations in the United Kingdom, Australia, Canada, and the United States. However, research on the frequency of *MUTYH* carriers in other populations remains limited (5, 6).

The MAP phenotype includes multiple adenomatous and serrated polyps, as well as mixed polyps (hyperplastic and adenomatous), with an increased lifetime risk of CRC and other extracolonic manifestations, including gastric, duodenal adenomas, and ovarian, bladder, breast, and endometrial cancers (7).

Clinically and histopathologically, MAP presents a variable phenotype that can overlap with the phenotype of classic familial adenomatous polyposis (FAP) (4), attenuated familial adenomatous polyposis (AFAP) and Lynch Syndrome. In all cases, there is a high risk of colon cancer, with an early onset of the disease (40–55 years old), usually in the proximal or ascending colon, and accompanied by a high number of infiltrating lymphocytes (8). However, the molecular mechanisms differ, leading to different medical management and genetic counseling approaches.

The diagnosis of MAP has important implications for clinical management. Current international guidelines recommend high-quality colonoscopic surveillance beginning no later than 25–30 years of age, with examinations every 1–2 years and endoscopic polypectomy whenever feasible. Surgical management, including colectomy with ileorectal anastomosis or proctocolectomy, should be considered when the adenoma burden is no longer amenable to endoscopic control, when advanced histology is identified, or when colorectal cancer develops. Because duodenal polyposis and duodenal cancer also occur in patients with MAP, baseline upper gastrointestinal endoscopy with complete visualization of the ampulla of Vater is recommended starting at 30–35 years of age, with subsequent surveillance according to duodenal findings. Annual physical examination is advised as part of extracolonic surveillance. Importantly, these recommendations apply to individuals with biallelic pathogenic *MUTYH* variants, whereas monoallelic carriers should generally follow population-based colorectal cancer screening recommendations unless modified by their personal or family history, highlighting the importance of accurate molecular diagnosis for appropriate surveillance and genetic counseling (9).

In this study, we characterized the clinical, histopathological phenotype (including colonic and extracolonic manifestations) and genotype of Colombian individuals with polyposis syndromes with a focus on *MUTYH*-associated polyposis. These cases were identified through the CHIBCHA project (Genetic study of Common Hereditary Bowel Cancers in Hispania and the Americas). Our findings contribute to a better understanding of the genetic architecture of CRC in underrepresented populations, as well as to improved risk stratification and genetic counseling strategies.

## 2 Case report

Three main cases with complete histopathological data and detailed family history were included in this report: two cases carrying the c.452A>G variant and one case carrying the c.1353_1355del variant. Family history information for the latter case was further expanded using the public database FamilySearch, and informed consent was obtained from all participants. Additionally, independent findings from Clínica Internacional de Alta Tecnología en Cáncer (Clinaltec) identified two further individuals carrying c.452A>G and one heterozygous carrier of c.1353_1355del detected during predictive multigene panel testing.

### 2.1 Case 1

The first case is a 32-year-old male diagnosed with a moderately differentiated, low-grade adenocarcinoma with a mucinous component located at the splenic flexure of the colon, classified as stage IIA. The patient underwent a right radical colectomy with ileotransversostomy. A total of 47 lymph nodes were examined, of which four were positive for carcinoma. Immunohistochemical (IHC) analysis revealed microsatellite instability (MSI-positive) and loss of MLH1 protein expression.

Over a 13-year follow-up period, the patient has undergone seven resections of tubular and tubulovillous adenomas with high-grade dysplasia, the most recent in 2011. Histopathological diagnoses included tubulovillous adenomas with high-grade dysplasia in the transverse and left colon, as well as a rectal polyp consistent with a tubulovillous adenoma containing intramucosal carcinoma.

The patient underwent germline genetic testing using a customized probe panel on the Fluidigm Access Array™ platform, followed by sequencing with the TruSight Cancer Panel on the Illumina MiSeq platform (**Supplementary Table 1**).

**Figure 1:**
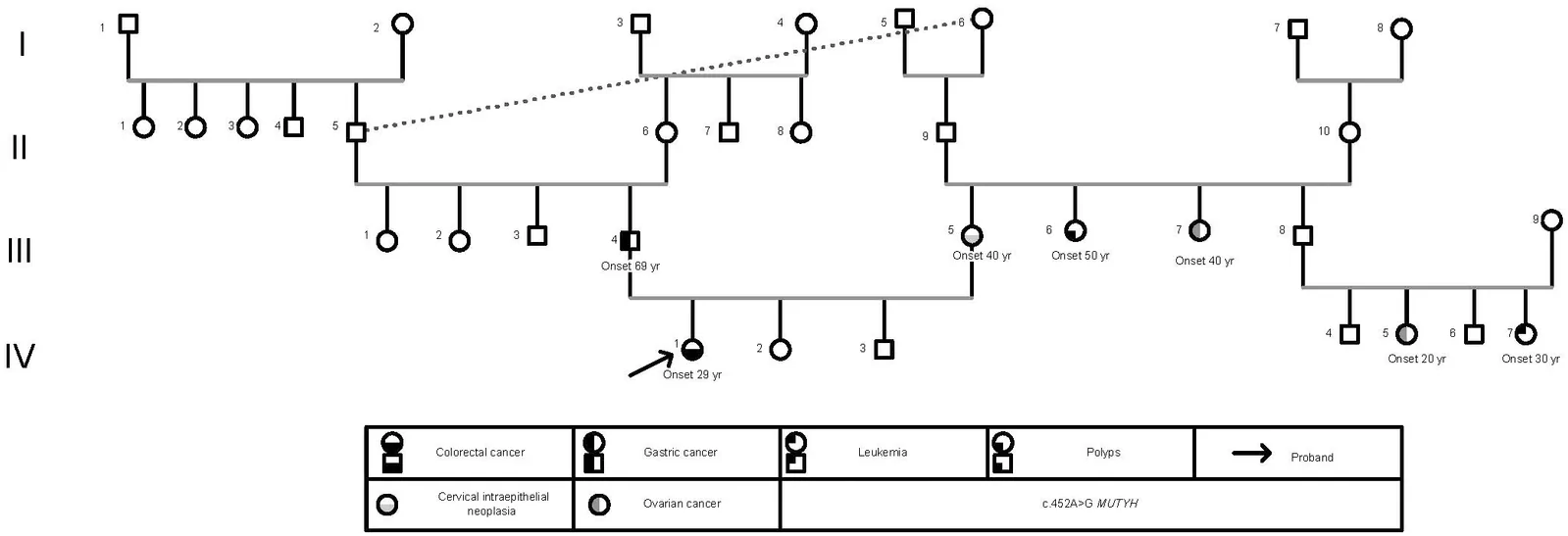
Pedigree of the family carrying the *MUTYH* variant *c.1353_1355del.* Circles represent females and squares represent males. Individuals with available biological samples are labeled with their age at interview, and the age at disease onset is indicated when applicable. CRC cases are shown with a filled middle-left quadrant, and individuals with an unknown cancer diagnosis are represented with a filled middle-right quadrant. Homozygous and heterozygous carriers of the *MUTYH* variant are indicated according to the legend. The proband is marked with an arrow.

Although no relevant personal or family history of malignancy was initially reported, this study validated the segregation of the identified mutation and confirmed that both parents and two sons carry the same mutation in heterozygosity. Additionally, the patient’s asymptomatic sister was found to carry a bi-allelic homozygous mutation (**Figure 1**).

The variant NM_001048174.2(*MUTYH*):c.1353_1355del p.(Glu452del) (rs587778541), also known as NM_001128425.2:c.1437_1439del p (Glu480del), was identified in this patient in homozygous state. This variant is located in exon 14 within the NUDIX domain and consists of a 3-nucleotide deletion, resulting in the loss of glutamic acid at position 452 of the polypeptide chain. The mutation has previously been reported in research studies and clinical databases as pathogenic in association with the MAP phenotype, both in homozygosity and in compound heterozygosity with other pathogenic variants (ClinVar ID: 127838) (10, 11).

In the gnomAD v3.1.2 population database (non-cancer), this variant was identified in heterozygosity in 8 individuals, of whom 7 correspond to the European (non-Finnish) population, out of a total of 147,874 individuals. Notably, it has not been described in homozygosity, and has been shown to segregate with the disease primarily in European populations (12–14). Furthermore, various studies using *in vitro* and cell culture models have demonstrated the deleterious effect of this variant on the enzymatic activity of the protein (15–17). Finally, based on this evidence and in accordance with the ACMG clinical guidelines for variant classification, this variant was classified as pathogenic, confirming the molecular diagnosis of MAP in the patient, with an autosomal recessive inheritance pattern.

This case was initially reported in Genetics of colorectal carcinoma with familial aggregation in a sample from the Colombian Andean Region (18).

### 2.2 Case 2

The case report is a 29-year-old female diagnosed with a malignant epithelial neoplasm, infiltrating moderately differentiated adenocarcinoma with lymphovascular invasion. Her past medical history reported chronic gastritis associated with *Helicobacter pylori* infection and a history of tobacco use for approximately 10 years. The clinical history revealed a 2.5-year history of intestinal pain. Additionally, the patient reported rectal bleeding for four months prior to diagnosis. A tumor was identified in the transverse colon, and the presence of polyps in the sigmoid colon was also documented.

Histopathological evaluation demonstrated a malignant epithelial neoplasm composed of closely packed tubular glandular structures, consistent with a moderately differentiated infiltrating adenocarcinoma, with evidence of lymphovascular invasion. Additional biopsy findings included a tubulovillous adenoma with low-grade dysplasia in the sigmoid colon. A mucosal biopsy from the upper rectum also revealed a moderately differentiated infiltrating adenocarcinoma with lymphovascular invasion.

**Figure 2:**
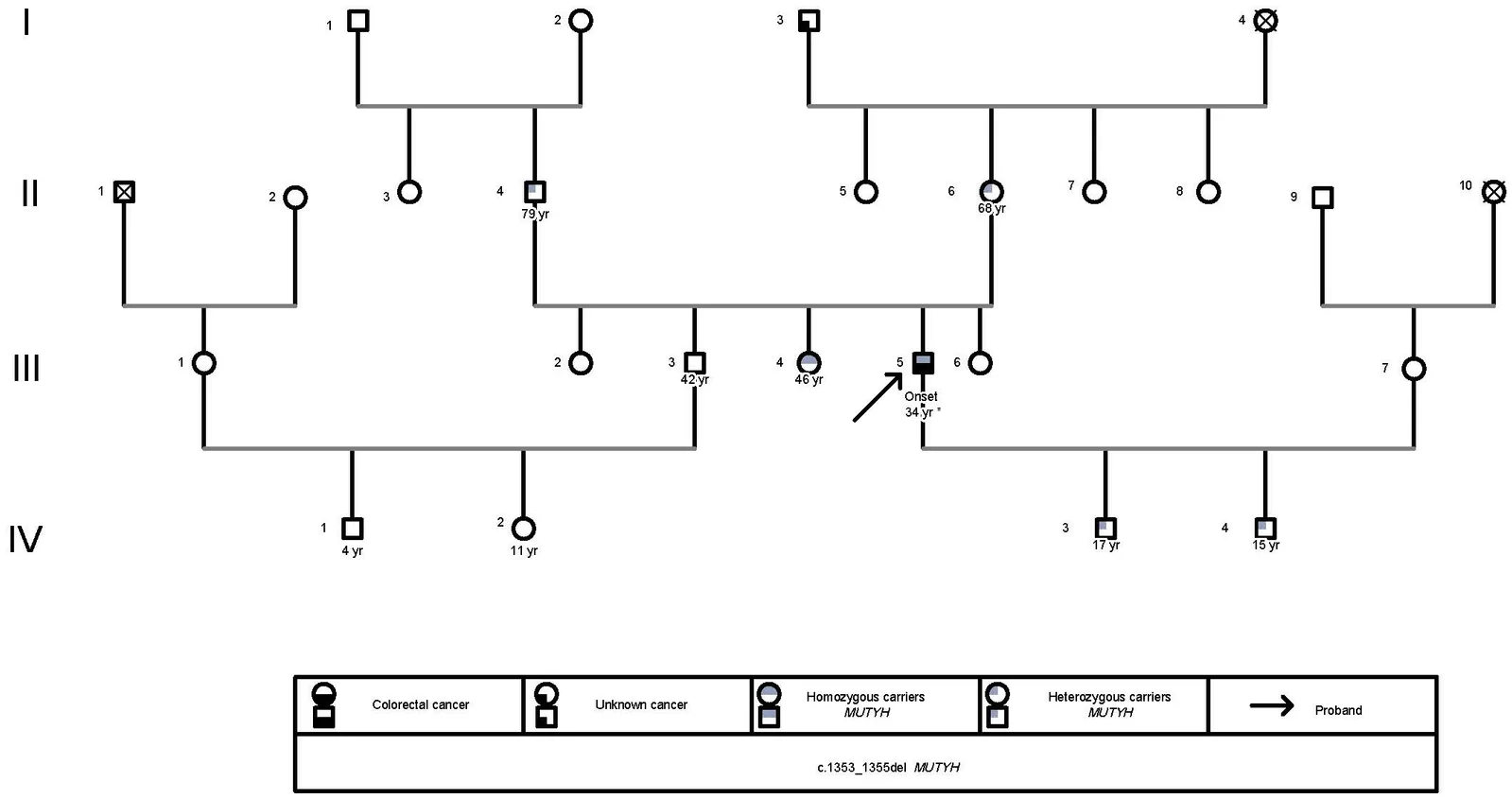
Pedigree of the case 2, carrying the *MUTYH* variant c.452A>G. Circles represent females and squares represent males. Colorectal cancer cases are represented with a filled middle-left quadrant, gastric cancer with a filled middle-right quadrant, leukemia with a filled upper-right quadrant, and polyps with a filled lower-left quadrant. Additional reported diagnoses, including cervical intraepithelial neoplasia and ovarian cancer, are indicated according to the legend. The proband is marked with an arrow. The dashed line between I-6 and II-5 denotes a reported kinship relationship between individuals from different branches of the family, as suggested by the proband.

The family history was significant for multiple malignancies, including colorectal, gastric, ovarian, and hematological cancers. The patient’s father had gastric adenocarcinoma, and her mother was diagnosed with cervical intraepithelial neoplasia grade II (CIN II) at the age of 40. One aunt developed ovarian cancer at age 40, while another aunt had a history of colon polyps. A cousin died from leukemia at the age of 30, and another cousin developed an ovarian tumor at the age of 20 (**Figure 2**).

The patient underwent germline genetic testing using the TruSight Hereditary Cancers Panel, followed by sequencing on the Illumina MiSeq platform (**Supplementary Table 1**).

Missense variant NM_001048174.2:c.452A>G (p.Tyr151Cys) (rs34612342) in *MUTYH*, also known as (NM_001128425.2:c.536A>G p.Try179Cys), was identified in the patient in heterozygosis. This variant consists of an amino acid substitution from a Tyrosine to a Cysteine at position 151 of the polypeptide chain located in the HhH-GPD domain, and has previously been reported as a common pathogenic variant in homozygosity and in compound heterozygosity state in multiple individuals with MAP and CRC (ClinVar ID: 5293) (2, 19).

Additionally, according to the latest ACMG guidelines, this variant (rs34612342) is considered as pathogenic, confirmed across different in-silico pathogenicity prediction tools (SIFT, PolyPhen2, AlphaMissense, REVEL) as well as *in vitro* studies models (20, 21). In gnomAD ver3.1.2 (non-cancer) tool, the variant was found in 229 (40 corresponding to Admixed American) out of 147,924 individuals. Segregation analysis could not be performed, as the proband’s relatives declined to participate in the study.

### 2.3 Case 3

The third case involves a 79-year-old female who was initially diagnosed at age 73 with a moderately differentiated adenocarcinoma, histological grade 2, exhibiting an intestinal-type moderately differentiated pattern. Clinical history evaluation revealed a neoplastic lesion in the sigmoid colon. Histopathological analysis demonstrated a malignant epithelial neoplasm with villous architecture and glandular formations composed of hyperchromatic and pleomorphic cells, leading to a final diagnosis of infiltrating adenocarcinoma.

**Figure 3:**
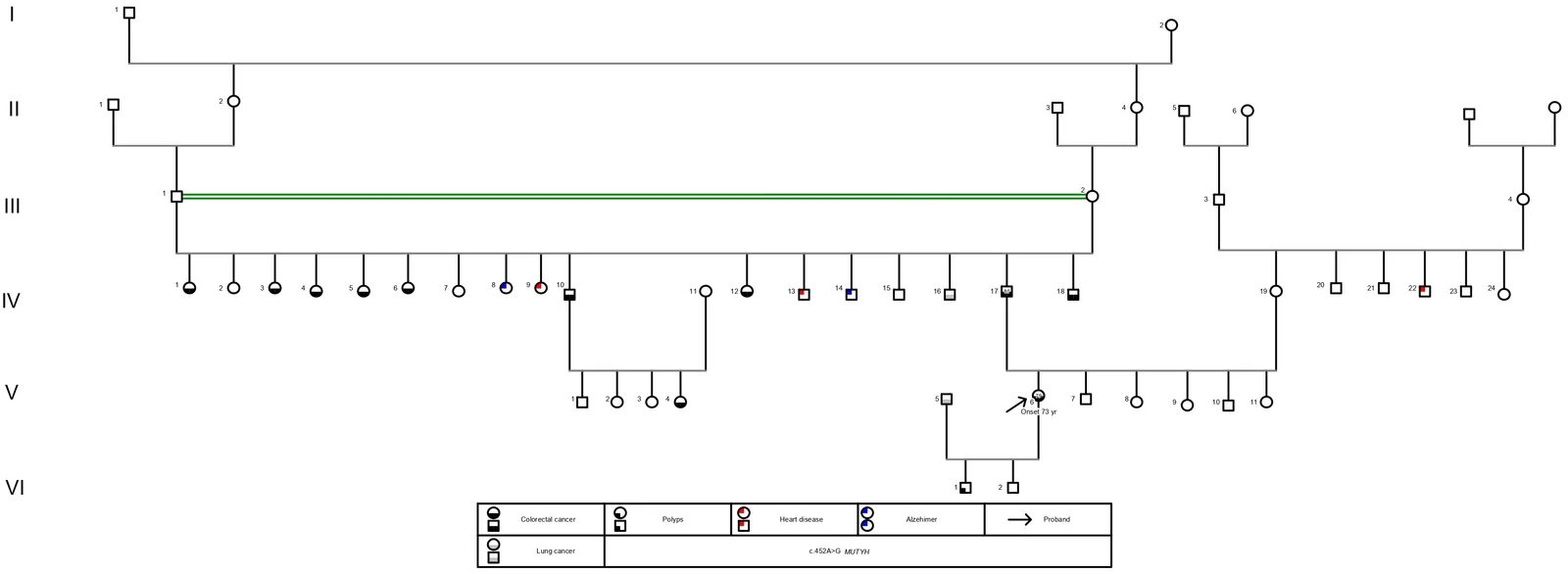
Pedigree of the case 3, carrying the *MUTYH* variant c.452A>G. Circles represent females and squares represent males. Cancer diagnoses are indicated according to the legend: colorectal cancer is shown with a filled middle-left quadrant and lung cancer with a filled lower-left quadrant. Individuals with polyps are indicated with a filled upper-left quadrant. Comorbidities are represented with colored symbols, including heart disease (red) and Alzheimer’s disease (blue). The proband is indicated by an arrow. The double green line indicates a consanguineous relationship.

The family history is significant for multiple malignancies and other conditions. The patient’s father died at 80 years-old from CRC and extensive history of the disease was identified among other relatives, including five aunts, two uncles, and one cousin affected. One paternal uncle was diagnosed with lung cancer. Neurodegenerative and cardiovascular conditions were also reported within the family, including Alzheimer’s disease in one uncle and one aunt, and documented heart disease in another uncle and aunt (**Figure 3**). The patient also reported a consanguineous relationship between her paternal grandparents, who were first cousins. Additionally, her son reported the presence of polyps. Germline genetic analysis was performed using the SureSelect XT HS2 DNA target enrichment system (Agilent^®^). Libraries were prepared according to the manufacturer’s protocol and sequenced on the DNBSEQ-G400 platform (MGI). The missense variant NM_001048174.2:c.452A>G (p.Tyr151Cys) (rs34612342) in *MUTYH*, was identified in this patient in the heterozygous state, as also observed in case 2.

### 2.4 Additional findings (Clinaltec retrospective screening)

This study also comprised a retrospective analysis of cancer patients or unaffected individuals with a family history of cancer, referred for germline genetic testing at Clínica Internacional de Alta Tecnología en Cáncer (Clinaltec), between September 2022 to December 2025. All patients provided written informed consent, following locally approved ethical protocols. This analysis identified one heterozygous carrier of the *MUTYH* c.1353_1355del variant and two additional carriers of the *MUTYH* c.452A>G variant. The two individuals referred by Clinaltec who carried the c.452A>G variant underwent whole-exome sequencing (WES) using the SureSelect Human All Exon V6 kit (Agilent Technologies), followed by sequencing on the Illumina NovaSeq 6000 platform (Illumina, Inc.). The c.1353_1355del variant carrier was analyzed using a custom 548-gene panel, with library preparation, target capture, sequencing, and primary bioinformatics processing performed by Novogene Inc. (Sacramento, CA, USA) according to the vendor’s standard protocols (**Supplementary Table 1**). In this case, the variant was an incidental finding unrelated to the indication for the multigene panel test.

Among the c.452A>G carriers, a 23-year-old female heterozygous was identified as a heterozygous carrier with a mature cystic teratoma in the ovary and chronic quiescent gastritis, she reported a family history of cancer and a history of uterine/ovarian cancer in her mother at age 47. Trio-exome sequencing confirmed paternal heterozygosity for the same variant, while her mother tested negative. A second individual was a 57-year-old female patient with colon cancer and an extensive colonic polyposis phenotype with more than 100 adenomatous polyps, predominantly villous, several of which presented high-grade dysplasia (Carcinoma *In Situ*). Tumor testing revealed the c.452A>G variant with 100% allelic balance, suggesting possible germline homozygosity, which requires confirmatory germline testing. She also reported a family history of CRC in three paternal uncles.

Additionally, a 48-year-old cancer-free female heterozygous carrier of c.1353_1355del variant, presented with a left breast intracanalicular fibroadenoma and a first-degree family history of breast cancer.

## 3 Discussion

*MUTYH*-associated polyposis (MAP) remains an under-recognized hereditary CRC syndrome, particularly in settings where genetic testing is not routinely available (22). Beyond the lack of reporting, our findings highlight an even more critical deficiency: there is still low clinical suspicion, insufficient use of a detailed family history in routine practice, and limited access to genetic counseling services.

In the three cases presented, extended pedigree reconstruction was essential to identify patterns of familial aggregation not initially evident, reinforcing that, in resource-limited contexts, systematic family history assessment remains a key tool to guide molecular testing. This strategy proved valuable for identifying familial aggregation patterns not initially evident in routine clinical interviews. In resource-limited contexts, systematic pedigree reconstruction may represent a feasible and cost-effective approach to prioritize genetic testing.

A key clinical implication is the distinction between biallelic pathogenic variants in *MUTYH*, which define MAP and confer a high risk of CRC, and monoallelic (heterozygous) carriers, whose risk is considerably lower. Misinterpretation of this difference may lead to inappropriate management, including over-surveillance or missed diagnosis (22).

The identification of the c.1353_1355del variant (p.Glu452del) in case 1 and in additional findings from the Clinaltec report contributes to the scarce data available in Latin American populations and underscores the need to better characterize the regional mutational spectrum.

On the other hand, the missense variant c.452A>G (p.Tyr151Cys) was detected in heterozygous state in cases 2 and 3, in addition to being reported in three individuals from Clinaltec. Three carriers presented clinical features, including invasive adenocarcinoma, and all reports coincide in having a relevant family history of cancer. c.452A>G variant has been reported in the heterozygous state in almost 2% of individuals of European ancestry (23) and in ~80% of MAP cases in populations with this ancestry (2).

These findings are consistent with the most recent extensive characterization of pathogenic germline variants of *MUTYH* in Latin America, where the *MUTYH*:c.452A>G variant has been reported in 24 of 105 patients with polyposis and/or colorectal cancer (24), in 1 of 119 patients with breast, ovarian or prostate cancer in a Uruguayan cohort (25), and in 1 of 17,554 cases of gnomAD v.2 (26). Furthermore, this variant has been described in a Colombian individual from a cohort of 222 patients from the Latin American and Caribbean (LACAM) population with suspected hereditary predisposition to breast and ovarian cancer (27), and in 10 individuals diagnosed with breast, ovarian, or colorectal cancer (one homozygous and nine heterozygous) in a Colombian cohort of 8165 patients with suspected hereditary cancer predisposition (28).

In this study, biallelic carriers frequently presented with severe polyposis phenotypes, including cases with more than 100 adenomatous polyps (18%), and colorectal cancer was the predominant neoplasm (75%). Additionally, a family history of colorectal cancer was reported, reinforcing the relevance of systematic evaluation of family history as a key clinical screening tool (24). This is consistent with a previous report from Colombia in which, among 11 index patients, all presented monoallelic variants of *MUTYH* and reported relevant family history of cancer, including prostate, colorectal, melanoma, breast, uterine and stomach cancer (29). While the potential association between mono-allelic *MUTYH* mutations and an elevated risk of extra-colonic cancers such breast, ovarian, prostate, bladder, duodenal or skin cancers remains controversial, these findings highlight the necessity for a careful interpretation when assessing the role of *MUTYH* mutations in cancer risk (30).

The recurrence of these *MUTYH* variants in Colombian individuals may reflect a shared demographic history and population admixture across the region. This interpretation is consistent with the complex tri-hybrid ancestry structure of Colombian populations, shaped by European, Native American, and African contributions (31). Particularly given that most pathogenic *MUTYH* variant reports have been described in European populations. This hypothesis is consistent with previous evidence of European founder mutations in Colombia, such as the *BRCA1*:c.3331_3334del variant reported in families from the Tolima–Huila region (Tolima Grande) (32, 33). These findings support the need for ancestry-informed and cost-effective genetic screening strategies to strengthen hereditary colorectal cancer prevention programs.

From a public health perspective, these findings highlight structural gaps in hereditary CRC prevention in underrepresented populations, including fragmented cancer registries and the limited integration of family history and genetic data. Strengthening these systems would improve risk stratification and enable targeted prevention strategies.

## 4 Conclusion

This study reports the first cases in ¨Tolima Grande¨ - Colombia of polyposis associated with *MUTYH* c.1353_1355del and c.452A>G variants and highlights its relevance in Latin populations. Accurate diagnosis requires integrating genetic testing with detailed family history and clear distinction between biallelic disease and heterozygous carriers. Limited access to genomic tools and underrepresentation in databases may lead to underdiagnosis. Strengthening clinical awareness and population-based registries to improve hereditary CRC risk stratification.

## Data Availability

The original contributions presented in the study are included in the article. In addition, inquiries can be directed to the corresponding author.
