# Supplementary material for: First report of MUTYH-associated polyposis with c.1353_1355del and c.452A>G mutations in Tolima Grande region from Colombia: a case report
Source: Front Oncol. 2026 Aug 17;16:1873371. doi: 10.3389/fonc.2026.1873371 (PMC13527933; doi:10.3389/fonc.2026.1873371)
Supplement: Supplementary file 1 [file DataSheet1.pdf]

Supplementary material

Supplementary table 1. Gene panels and sequencing platforms were used for the molecular characterization of the reported cases.

The table details the sequencing platforms and the genes included in the hereditary cancer panels used for germline analysis in each reported case and in the additional individuals identified through retrospective screening at Clinaltec. Depending on the assay, the panels included the analysis of SNVs, INDELs, CNVs/CNAs, and gene fusions

**CASE 1**							
NM_001048174.2(MUTYH):c.1353_1355del p.(Glu452del)							
**Genes covered of SNV/INDEL (Fluidigm's Access Array™)**							
APC	*KRAS*	*MLH1*	*MSH2*	*MSH6*	*MUTYH*	*PMS2*	*POLE*
POLD1	*PTEN*	*STK11*	*TP53*				
**Genes covered of SNV/INDEL (TruSight Cancer Panel -Illumina)**							
ABL1	*AKT1*	*ALK*	*APC*	*ATM*	*BRAF*	*CDH1*	*CDKN2A*

CSF1R	*CTNNB1*	*EGFR*	*ERBB2*	*ERBB4*	*EZH2*	*FBXW7*	*FGFR1*
FGFR2	*FGFR3*	*FLT3*	*GNA11*	*GNAQ*	*GNAS*	*HNF1A*	*HRAS*
IDH1	*IDH2*	*JAK2*	*JAK3*	*KDR*	*KIT*	*KRAS*	*MET*
MLH1	*MPL*	*NOTCH1*	*NPM1*	*NRAS*	*PDGFRA*	*PIK3CA*	*PTEN*
PTPN11	*RB1*	*RET*	*SMAD4*	*SMARCB1*	*SMO*	*SRC*	*STK11*
TP53	*VHL*						
**CASE 2**							
**NM_001048174.2:c.452A>G (p.Tyr151Cys) (rs34612342)**							
**Genes covered of SNV/INDEL (TruSight Hereditary Cancers panel – Illumina®)**							

APC	*ATM*	*AXIN2*	*BARD1*	*BMPR1A*	*BRCA1*	*BRCA2*	*BRIP1*
CDH1	*CDK4*	*CDKN2A (p14ARF)*	*CDKN2A (p16INKa)*	*CHEK2*	*CTNNA1*	*DICER1*	*EPCAM*
GREM	*GREM1*	*KIT*	*MEN1*	*MLH1*	*MSH2*	*MSH3*	*MSH6*
MUTYH	*NBN*	*NF1*	*PALB2*	*PDGFRA*	*PMS2*	*PLD1*	*POLE*
PTEN	*RAD50*	*RAD52D*	*SDHB*	*SDHC*	*SDHB*	*SMAD4*	*SMARCA4*
STK11	*PT53*	*TSC1*	*TSC2*	*VHL*			
**CASE 3**							
NM_001048174.2:c.452A>G (p.Tyr151Cys) (rs34612342)							
**Genes covered of SNV/INDEL/CNVs (Sureselect XT HS2 DNA – Agilent®)**							

APC	*AXIN2*	*BMPR1A*	*BRCA1*	*BRCA2*	*CDH1*	*CHEK2*	*EPCAM*
GREM1	*MLH1*	*MSH2*	*MSH3*	*MSH6*	*MUTYH*	*NTHL1*	*PMS2*
POLD1	*POLE*	*PTEN*	*SMAD4*	*STK11*	*TP53*		
**Additional findings (Clinaltec retrospective screening)**							
**Carrier of the c.1353_1355del variant**							
**Genes covered of SNV/INDEL/CNAs**							
ABCB1	*ABCC1*	*ABCC2*	*ABCC3*	*ABCC4*	*ABCC6*	*ABCG2*	*ABL1*
ABL2	*ACTG1*	*ACVR1B*	*ACVR2A*	*AKT1*	*AKT2*	*AKT3*	*ALK*
AMER1	*APC*	*AR*	*ARAF*	*ARFRP1*	*ARID1A*	*ARID1B*	*ARID2*
ASXL1	*ATIC*	*ATM*	*ATR*	*ATRX*	*AURKA*	*AURKB*	*AXIN1*

AXIN2	*AXL*	*BAP1*	*BARD1*	*BAX*	*BCL10*	*BCL11A*	*BCL2*
BCL2L1	*BCL2L2*	*BCL6*	*BCOR*	*BCORL1*	*BCR*	*BIRC5*	*BLCAP*
BLK	*BLM*	*BMPR1A*	*BRAF*	*BRCA1*	*BRCA2*	*BRD3*	*BRD4*
BRIP1	*BTG1*	*BTK*	*C11orf30*	*C17orf108*	*C8orf34*	*CAMK2G*	*CAMKK2*
CARD11	*CASP7*	*CASP8*	*CBFB*	*CBL*	*CBR1*	*CBR3*	*CCND1*
CCND2	*CCND3*	*CCNE1*	*CD19*	*CD22*	*CD274*	*CD33*	*CD52*
CD74	*CD79A*	*CD79B*	*CDA*	*CDC25C*	*CDC42*	*CDC73*	*CDH1*
CDK1	*CDK12*	*CDK2*	*CDK4*	*CDK5*	*CDK6*	*CDK7*	*CDK8*
CDK9	*CDKN1A*	*CDKN1B*	*CDKN2A*	*CDKN2B*	*CDKN2C*	*CEBPA*	*CHD2*
CHD3	*CHD4*	*CHEK1*	*CHEK2*	*CIC*	*CNTNAP1*	*CNTNAP2*	*COL22A1*

COMT	*COPS3*	*CREBBP*	*CRKL*	*CRLF2*	*CSF1R*	*CSMD1*	*CSMD3*
CTCF	*CTLA4*	*CTNNA1*	*CTNNB1*	*CUL3*	*CXCL10*	*CXCL8*	*CXCR4*
CYLD	*CYP19A1*	*CYP1A1*	*CYP1A2*	*CYP1B1*	*CYP2A6*	*CYP2B6*	*CYP2C19*
CYP2C8	*CYP2C9*	*CYP2D6*	*CYP2E1*	*CYP3A4*	*CYP3A5*	*CYP4B1*	*DAXX*
DDR2	*DHFR*	*DICER1*	*DIRAS3*	*DNMT3A*	*DOT1L*	*DPYD*	*DYNC2H1*
E2F1	*EGF*	*EGFR*	*EML4*	*ENOSF1*	*EP300*	*EPCAM*	*EPHA3*
EPHA5	*EPHA7*	*EPHB1*	*EPHX1*	*ERBB2*	*ERBB3*	*ERBB4*	*ERCC1*
ERCC2	*ERCC4*	*ERG*	*ERRFI1*	*ESR1/ER*	*ETV1*	*ETV4*	*ETV5*
ETV6	*EWSR1*	*EXT1*	*EXT2*	*EZH2*	*FAM46C*	*FANCA*	*FANCC*
FANCD2	*FANCE*	*FANCF*	*FANCG*	*FANCL*	*FAS*	*FAT1*	*FBXW7*

FCGR3A	*FGF10*	*FGF14*	*FGF19*	*FGF23*	*FGF3*	*FGF4*	*FGF6*
FGFR1	*FGFR2*	*FGFR3*	*FGFR4*	*FH*	*FLCN*	*FLT1*	*FLT3*
FLT4	*FOLR3*	*FOXA1*	*FOXA2*	*FOXL2*	*FOXO1*	*FOXP1*	*FRS2*
FUBP1	*FYN*	*GABRA6*	*GATA1*	*GATA2*	*GATA3*	*GATA4*	*GATA6*
GGH	*GID4*	*GLI1*	*GLI2*	*GNA11*	*GNA13*	*GNAQ*	*GNAS*
GOPC	*GPR124*	*GRB2*	*GRIN2A*	*GRM3*	*GSK3B*	*GSTA1*	*GSTM3*
GSTP1	*H3F3A*	*HDAC1*	*HDAC2*	*HDAC3*	*HDAC4*	*HDAC6*	*HDAC8*
HGF	*HIF-1/HIF1A*	*HNF1A*	*HRAS*	*HSD3B1*	*HSP90AA1/HSP90*	*IDH1*	*IDH2*
IGF-1	*IGF1R/IGFR*	*IGF2*	*IGF2R*	*IKBKB*	*IKBKE*	*IKZF1*	*IL7R*
INHBA	*INPP4B*	*IRF2*	*IRF4*	*IRS2*	*ITK*	*JAK1*	*JAK2*

JAK3	*JUN*	*KAT6A*	*KDM5A*	*KDM5C*	*KDM6A*	*KDR/VEGFR*	*KEAP1*
KEL	*KIAA0427*	*KIT*	*KLHL6*	*KMT2A/MLL*	*KMT2B*	*KMT2C/MLL3*	*KMT2D/MLL2*
KRAS	*LMO1*	*LRP1B*	*LRRK2*	*LYN*	*LZTR1*	*MAGI2*	*MAP2K1/MEK1*
MAP2K2	*MAP2K4*	*MAP3K1*	*MAP4K1*	*MAPK1*	*MAPK14*	*MAPK8*	*MAPK9*
MCL1	*MDM2*	*MDM4*	*MED12*	*MEF2B*	*MEN1*	*MET*	*MITF*
MLH1	*MMP12*	*MMP14*	*MMP9*	*MPL*	*MRE11A*	*MSH2*	*MSH6*
MTHFR	*MTOR*	*MTR*	*MUTYH*	*MYB*	*MYC*	*MYCL*	*MYCN*
MYD88	*MYO1B*	*NAT1*	*NAT2*	*NBN*	*NCOR1*	*NF1*	*NF2*
NFE2L2	*NFKBIA*	*NKX2-1*	*NOTCH1*	*NOTCH2*	*NOTCH3*	*NOTCH4*	*NPM1*
NRAS	*NRG1*	*NSD1*	*NTRK1*	*NTRK2*	*NTRK3*	*NUP93*	*OPRM1*

PAK1	*PAK3*	*PALB2*	*PARK2*	*PARP1*	*PARP2*	*PARP3*	*PARP4*
PAX5	*PBRM1*	*PDCD1*	*PDCD1LG2*	*PDGFRA*	*PDGFRB*	*PDK1*	*PEG3*
PIK3C2B	*PIK3CA*	*PIK3CB*	*PIK3CG*	*PIK3R1*	*PIK3R2*	*PKC/PRRT2*	*PKCγ*
PKCε/PRKCE	*PLCG2*	*PLK1*	*PMS1*	*PMS2*	*POLD1*	*POLE*	*PPARG*
PPP2R1A	*PPP2R5B*	*PRDM1*	*PREX2*	*PRKAR1A*	*PRKCI*	*PRKDC*	*PRSS8*
PTCH1	*PTCH2*	*PTEN*	*PTK2*	*PTPN11*	*QKI*	*RAC1*	*RAC2*
RAD50	*RAD51*	*RAD51C*	*RAD51D*	*RAF1*	*RANBP2*	*RARA*	*RARB*
RASSF1	*RASSF8*	*RB1*	*RBM10*	*REL*	*RET*	*RHEB*	*RHOA*
RICTOR	*RNF43*	*ROCK1*	*ROS1*	*RPS6KA1*	*RPS6KB1*	*RPTOR*	*RRM1*
RUNX1/AML1	*RUNX1T1*	*RUNX2*	*SATB2*	*SDHA*	*SDHB*	*SDHC*	*SDHD*

SETD2	*SF3B1*	*SHH*	*SLC10A2*	*SLC16A7*	*SLC19A1*	*SLC22A16*	*SLC28A3*
SLCO1B3	*SLIT2*	*SMAD2*	*SMAD3*	*SMAD4*	*SMARCA1*	*SMARCA4*	*SMARCB1*
SMARCD1	*SMO*	*SNAI1*	*SNAI2*	*SNCAIP*	*SOCS1*	*SOD2*	*SOX10*
SOX17	*SOX2*	*SOX9*	*SPEN*	*SPOP*	*SPTA1*	*SRC*	*STAG2*
STAT3	*STAT4*	*STK11*	*STK4*	*SUFU*	*SYK*	*TAF1*	*TBX3*
TCF7L2	*TEK*	*TERT*	*TET2*	*TGFBR1*	*TGFBR2*	*TLR4*	*TMPRSS2*
TNF/TNF-alpha	*TNFAIP3*	*TNFRSF11A*	*TNFRSF14*	*TNFSF11*	*TOP1*	*TOP2A*	*TP53*
TPMT	*TSC1*	*TSC2*	*TSHR*	*TUBA1A*	*TUBB*	*TUBD1*	*TUBE1*
TWIST1	*TYMS/TS*	*U2AF1*	*UGT1A1*	*UGT1A9*	*UMPS*	*VEGFA*	*VEGFB*
VHL	*WEE1*	*WISP3*	*WNT1*	*WNT5A*	*WNT6*	*WT1*	*XIAP*

XPC	*XPO1*	*XRCC1*	*YES1*	*ZBTB2*	*ZNF217*	*ZNF703*	*AIP*
BUB1	*BUB1B*	*BUB3*	*CDKN1C*	*CEP57*	*DDB2*	*DIS3L2*	*ERCC3*
ERCC5	*FANCB*	*FANCI*	*FANCM*	*GPC3*	*HOXB13*	*MAX*	*MSH3*
PHOX2B	*PRF1*	*PRSS1*	*RECQL4*	*RHBDF2*	*SBDS*	*SDHAF2*	*SLX4*
SPINK1	*TMEM127*	*WRN*	*XPA*				
**Genes covered of fusions**							
ALK	*BCR*	*BRAF*	*BRD4*	*ETV1*	*ETV4*	*ETV5*	*ETV6*
EWSR1	*FGFR1*	*FGFR3*	*JAK2*	*KMT2A*	*MYB*	*NTRK1*	*NTRK2*
PPARG	*RAF1*	*RET*	*ROS1*	*TMPRSS2*			
